# The Role of Artificial Intelligence in Managing Bipolar Disorder: A New Frontier in Patient Care

**DOI:** 10.3390/jcm14072515

**Published:** 2025-04-07

**Authors:** Jelena Milic, Iva Zrnic, Edita Grego, Dragana Jovic, Veroslava Stankovic, Sanja Djurdjevic, Rosa Sapic

**Affiliations:** 1 Institute of Public Health of Serbia “Dr Milan Jovanović Batut”, Dr Subotića Starijeg 5, 11 000 Belgrade, Serbia; 2 European Faculty “Kallos”, Ratarski put 8a, 11 000 Belgrade, Serbia; 3 Regional Medical Chamber of Belgrade, 11 000 Belgrade, Serbia; 4 The College of Health Science, Academy of Applied Studies, 11 000 Belgrade, Serbia; 5 Academy for Human Development, 11 000 Belgrade, Serbia; 6 Faculty of Health Studies, University of Bjeljina, 76 300 Bjeljina, Bosnia and Herzegovina

**Keywords:** bipolar disorder, artificial intelligence, mood episode prediction, personalized treatment, digital health, real-time support

## Abstract

**Background/Objectives:** Bipolar disorder (BD) is a complex and chronic mental health condition that poses significant challenges for both patients and healthcare providers. Traditional treatment methods, including medication and therapy, remain vital, but there is increasing interest in the application of artificial intelligence (AI) to enhance BD management. AI has the potential to improve mood episode prediction, personalize treatment plans, and provide real-time support, offering new opportunities for managing BD more effectively. Our primary objective was to explore the potential role of AI in transforming the management of BD, specifically in mood tracking, prediction, and personalized treatment regimens. **Methods**: To explore the potential role of AI in transforming BD management, we conducted a review of recent literature using key search terms. We included studies that discussed AI applications in mood tracking, prediction, and treatment personalization. The studies were selected based on their relevance to AI’s role in BD management, with attention to the PICO criteria: Population—individuals diagnosed with BD; Intervention—AI tools for mood prediction, treatment personalization, and real-time support; Comparison—traditional treatment methods (when available); Outcome—measures of mood episode prediction, treatment effectiveness, and improvements in patient care. **Results**: The findings from recent research reveal promising developments in the use of AI for BD management. Studies suggest that AI-powered tools can enable more proactive and personalized care, improving treatment outcomes and reducing the burden on healthcare professionals. AI’s ability to analyze data from wearable devices, smartphones, and even social media platforms provides valuable insights for early detection and more dynamic treatment adjustments. **Conclusions**: While AI’s application in BD management is still in its early stages, it presents transformative potential for improving patient care. However, further research and development are crucial to fully realize AI’s potential in supporting BD patients and optimizing treatment efficacy.

## 1. Introduction

Bipolar disorder (BD) is a chronic and complex mental health condition that affects millions of people worldwide. Characterized by extreme mood swings, including manic and depressive episodes, BD disrupts daily life and creates significant challenges for individuals and healthcare providers alike. Traditional treatment strategies, such as medication and psychotherapy, have been effective in managing symptoms, but often fail to address the underlying causes of these mood fluctuations. Consequently, the mental health community has been exploring innovative ways to improve treatment efficacy and patient outcomes.

### 1.1. What Is Bipolar Disorder?

Bipolar disorder (BD) is a chronic and complex mental health condition that affects millions of people worldwide. Characterized by extreme mood swings, including manic and depressive episodes, BD disrupts daily life and creates significant challenges for individuals and healthcare providers alike. Bipolar disorder is characterized by dramatic and unpredictable shifts between manic and depressive states. During manic episodes, individuals may experience euphoric feelings, increased energy, racing thoughts, and impulsive behavior, which can result in reckless decision making. On the other hand, depressive episodes bring feelings of deep sadness, hopelessness, fatigue, and a loss of interest in daily activities. The fluctuating nature of the disorder creates challenges in care, as individuals may present very different needs depending on whether they are in a manic or depressive phase. Traditional treatment strategies, such as medication and psychotherapy, have been effective in managing symptoms, but often fail to address the underlying causes of these mood fluctuations. Consequently, the mental health community has been exploring innovative ways to improve treatment efficacy and patient outcomes. One such promising advancement is the application of artificial intelligence (AI) in managing BD. While traditional treatments for bipolar disorder (BD), including pharmacotherapy and psychotherapy, remain the foundation of care, AI-driven approaches offer complementary benefits by enhancing precision, early detection, and real-time monitoring. Unlike conventional methods, which often rely on retrospective assessments and patient self-reports, AI-powered tools analyze continuous behavioral, physiological, and linguistic data to identify subtle mood shifts before they escalate. Studies suggest that AI models can improve treatment personalization, optimize medication adjustments, and provide real-time therapeutic support through chatbots and digital interventions [1].

### 1.2. The Rise of AI in Mental Health: A Game-Changer for Bipolar Disorder Management

Artificial intelligence (AI) is revolutionizing healthcare by offering innovative solutions to diagnose, manage, and treat mental health disorders [2]. In the context of bipolar disorder (BD), AI facilitates continuous, data-driven care, empowering clinicians to predict mood fluctuations, tailor treatments, and deliver real-time support [3]. Furthermore, AI allows for the development of personalized treatment plans by analyzing genetic, lifestyle, and medical history factors, enhancing the management of BD through a more individualized and proactive approach [4]. Initially, AI has gained prominence in the realm of psychiatric conditions, such as eating disorders (EDs), where it enhanced the diagnostic accuracy by addressing the diverse manifestations of these disorders across individuals. This refinement in diagnostic precision helps reduce delays and misdiagnoses, thereby optimizing healthcare resource allocation [5]. According to Di Stefano et al. (2024) [6] and Pettorruso et al. [7], this study highlights the transformative impact of AI-enhanced fMRI in schizophrenia research and clinical practice. By integrating machine learning and deep learning techniques, including Vision Transformers and support vector machines, AI has significantly improved the precision of detecting neural abnormalities and identifying biomarkers, surpassing conventional methods. The combination of fMRI’s ability to reveal structural and functional brain disruptions with AI’s analytical power enables the development of personalized therapeutic strategies tailored to individual neural profiles [6]. Another approach that highlights the growing role of personalized, data-driven medicine in tailoring psychiatric interventions and advancing mental health care is the ESK-LEARNING study (Predicting Outcome with Intranasal Esketamine Treatment: A Machine-Learning, Three-Month Study in Treatment-Resistant Depression). It demonstrates the potential of machine learning in optimizing therapeutic strategies. By analyzing patient-specific data, AI-driven models enhance the ability to identify individuals most likely to benefit from intranasal esketamine, improving both treatment precision and clinical decision making [7].

**Figure 1:**
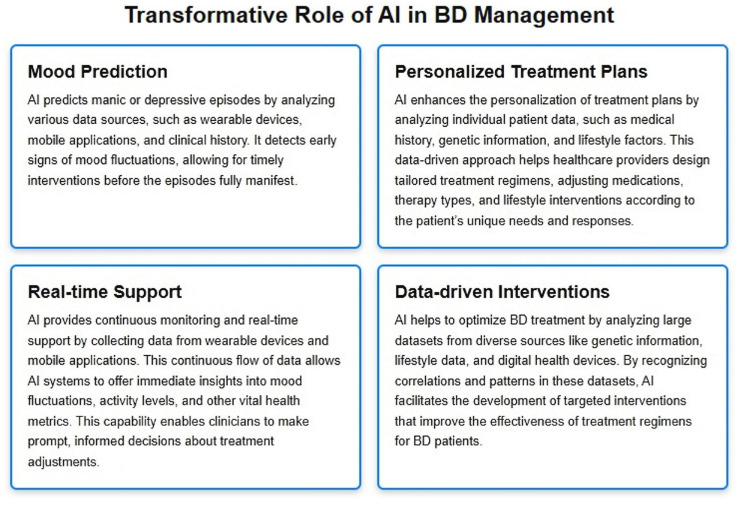
Transformative role of AI in BD management [4].

In BD management, traditional approaches rely on medication, therapy, and lifestyle adjustments, but often lack the predictive precision required to anticipate mood episodes. AI, however, harnesses data from sleep patterns, activity levels, and social media behavior to accurately forecast mood shifts, enabling early intervention. Furthermore, AI can craft personalized treatment plans by analyzing genetic, lifestyle, and medical history factors, leading to a more efficient and individualized approach to BD management. With AI, care for bipolar disorder shifts from a reactive framework to a proactive, tailored model. The potentials of AI to transform BD management are shown in Figure 1 [4].

This figure visually illustrates the transformative role of AI in the management of Bipolar Disorder (BD). AI plays a crucial part in enhancing treatment and care strategies by enabling mood prediction, personalized treatment plans, real-time support, and data-driven interventions.

### 1.3. AI-Powered Mood Tracking: Enhancing Real-Time Monitoring of Mood Fluctuation

A promising development in the management of bipolar disorder (BD) is the integration of artificial intelligence (AI) for real-time mood tracking and prediction. Traditional methods of monitoring mood fluctuations, primarily relying on self-reporting, are often subjective and inconsistent, limiting their effectiveness. For individuals with BD, early signs of mood changes can be subtle, making it challenging to recognize them before they escalate. AI-powered mood tracking systems offer a more objective alternative by leveraging data from wearable devices, smartphone apps, and other digital platforms. These systems can track a broad range of behaviors, such as sleep patterns, physical activity, speech speed, and social interactions, to detect early signs of mood shifts. Machine learning algorithms analyze these data to identify patterns and predict mood changes with increasing accuracy. By detecting early indicators of mania or depression, AI enables clinicians to intervene before episodes fully develop, thus preventing exacerbations and improving treatment outcomes. Furthermore, continuous monitoring provides patients with valuable insights into their mood cycles and triggers, allowing them to adjust behaviors, manage stress, and engage in coping strategies in real time. This shift from periodic check-ins to continuous monitoring makes AI-powered mood tracking a powerful tool in developing more personalized and effective treatment plans for individuals living with bipolar disorder [8].

### 1.4. Personalized Treatment Plans: AI’s Role in Tailoring Therapy to Individual Needs

Personalized medicine represents one of the most promising advancements in healthcare, and artificial intelligence (AI) plays a pivotal role in transforming this approach for bipolar disorder (BD). Unlike conventional, one-size-fits-all treatment strategies, personalized care considers an individual’s unique genetic makeup, lifestyle, behavior, and medical history. AI systems are capable of analyzing large volumes of data to create highly customized treatment plans for BD patients. For instance, machine learning algorithms can assess genetic predispositions to determine the most effective medications, minimizing trial-and-error in treatment. Furthermore, AI tracks patients’ responses to various therapies and makes real-time adjustments to optimize treatment, ensuring ongoing efficacy. By incorporating data such as sleep patterns, physical activity, and emotional responses, AI enables clinicians to refine treatment plans with greater precision. This individualized approach contrasts with traditional methods, which often rely on broad guidelines and patient self-reports. By integrating diverse data sources—genetics, behavior, and environmental factors—AI facilitates a more holistic and tailored strategy for BD management. Ultimately, AI-driven personalized treatment offers patients more effective, proactive care, enhancing their quality of life and reducing the frequency of manic and depressive episodes [9].

### 1.5. Chatbots for Emotional Support: Filling the Gap Between Therapy Sessions

Bipolar disorder (BD) often necessitates continuous therapeutic interventions to manage mood episodes effectively. However, significant gaps can arise between therapy sessions, leaving patients without immediate support during critical moments [10]. AI-powered chatbots and virtual assistants present an innovative solution to address this issue by providing real-time emotional support. These AI-driven tools simulate human interaction and offer a variety of coping strategies when patients experience manic or depressive episodes. For example, if a patient shows early signs of a manic episode, the chatbot can suggest relaxation techniques, mindfulness exercises, or guide them through cognitive reframing. During depressive episodes, the chatbot can provide emotional support, encourage healthy behaviors, and help patients stay connected to their treatment plan. The primary advantage of AI chatbots lies in their accessibility, allowing patients to access immediate assistance at any time, particularly when professional therapists or caregivers are unavailable. Additionally, these chatbots can track user interactions over time, providing valuable data that clinicians can use to refine treatment strategies. Moreover, AI chatbots offer a non-judgmental space for patients to express their thoughts and emotions, fostering a sense of comfort and confidentiality that might be challenging in human interactions. While AI chatbots are not a replacement for human therapy, they serve as an important complementary tool, providing timely emotional relief and support during critical moments [11,12].

### 1.6. AI in Cognitive Behavioral Therapy (CBT): Enhancing Traditional Approaches

Cognitive Behavioral Therapy (CBT) is a well-established treatment for bipolar disorder (BD), helping patients recognize and modify negative thought patterns that contribute to mood instability [13]. However, traditional CBT is typically delivered in structured sessions, leaving patients without immediate support during daily challenges. Artificial intelligence (AI) has the potential to enhance the CBT process by providing real-time, automated feedback and continuous support. AI-driven platforms allow patients to log their thoughts, behaviors, and emotional responses throughout the day, enabling the system to analyze patterns and detect cognitive distortions. These platforms offer immediate feedback and personalized suggestions, guiding patients in reframing negative thoughts and adopting more effective coping strategies. By bridging the gap between therapy sessions, AI-powered CBT ensures that patients remain engaged with their therapeutic goals while reinforcing positive behavioral changes. Additionally, AI can track progress over time, generating personalized insights that inform future therapy sessions and optimize treatment strategies. Integrating AI with traditional CBT offers a more dynamic and individualized approach, improving long-term treatment outcomes and providing patients with consistent, accessible mental health support beyond the therapy room [14].

### 1.7. Predictive Models for Relapse Prevention: AI as a Proactive Tool for Care

Relapse prevention is a major challenge in bipolar disorder (BD) management, with traditional care often responding only after episodes occur. Artificial intelligence (AI) offers a proactive solution by analyzing behavioral patterns—such as sleep, activity, and communication—to predict mood episodes before they escalate. Using data from wearable devices, smartphone apps, and social media, AI models detect early warning signs, enabling timely interventions such as medication adjustments or lifestyle changes. This predictive approach reduces the episode severity, prevents hospitalizations, and enhances treatment outcomes. Additionally, AI helps identify individual triggers, offering a more personalized and empowering approach to BD care. By shifting from reactive to proactive management, AI-driven models open new possibilities for relapse prevention and long-term stability [15,16].

### 1.8. Social Media Monitoring: Gaining Behavioral Insights Through AI Analysis

Social media serves as a key platform for individuals to express emotions, thoughts, and daily experiences. For patients with bipolar disorder (BD), analyzing social media activity can provide valuable insights into mood fluctuations. AI-powered systems track language, communication patterns, and behavioral shifts across platforms like Twitter, Facebook, and Instagram, detecting subtle signs of mood changes. Increased posting or shifts in tone may indicate mania, while withdrawal or negative language can signal depression. By processing vast amounts of data, AI provides real-time behavioral insights, enabling early intervention and personalized treatment adjustments. While privacy concerns must be considered, responsible AI-driven social media monitoring offers a powerful tool for detecting mood shifts and enhancing BD management [17,18,19].

### 1.9. Overcoming Challenges: Ethical and Practical Considerations in AI-Driven Mental Health

As AI continues to advance in bipolar disorder (BD) management, it raises important ethical and practical challenges. Privacy remains a key concern, requiring stringent data security measures and transparency in how patient information is collected, stored, and used. Informed consent is essential to ensure patients understand and control their data. Additionally, while AI enhances personalized care and predictive capabilities, it should complement rather than replace human interaction, as empathy and clinical judgment are irreplaceable in mental health care. Another critical challenge is accessibility—AI-driven tools must be inclusive and designed to serve diverse populations across different socioeconomic and cultural backgrounds. Addressing these issues is essential to ensuring AI’s ethical and effective integration into BD management, maximizing its benefits while safeguarding patient rights and well-being [20].

### 1.10. Research Gaps and Future Directions: The Need for Further Exploration

While AI is increasingly integrated into bipolar disorder (BD) management, further research is needed to fully understand its potential and limitations. Many AI-driven tools remain in early development or have been tested on limited populations, necessitating large-scale, longitudinal studies to validate their real-world effectiveness. Refining predictive algorithms is also essential to ensure accuracy and reliability across diverse BD patients. Additionally, research should explore how AI can best complement existing treatments, such as medication and psychotherapy, to enhance overall care. Ethical considerations, including patient privacy, consent, and safety, must also be prioritized. As AI-powered tools become more widespread, the ongoing evaluation of their long-term impact on mood tracking, relapse prevention, and therapy effectiveness will be crucial. With continued research and development, AI has the potential to significantly improve BD management, offering more personalized and proactive care [21,22].

### 1.11. Perspective: AI’s Transformative Potential in Bipolar Disorder Care

Artificial intelligence (AI) has the potential to revolutionize the treatment of bipolar disorder (BD) by enabling continuous mood tracking, predicting mood episodes, personalizing treatment plans, and providing real-time support through chatbots and virtual assistants. Its integration into BD care can enhance the diagnostic accuracy, optimize treatment strategies, and improve patient outcomes. However, challenges remain, particularly regarding ethical considerations, data privacy, and accessibility. Despite these obstacles, AI’s role in BD management is poised to expand as research advances and technologies become more sophisticated. With a balanced integration of AI and human care, BD treatment can become more personalized, proactive, and effective, ultimately improving patient well-being [19,23].

### 1.12. Objectives

Our primary objective was to explore the potential role of AI in transforming the management of BD. This includes investigating how AI tools, such as mood tracking devices and machine learning algorithms, could be used to monitor mood fluctuations in real-time, predict manic or depressive episodes, and develop personalized treatment regimens.

Our secondary objective was to review the findings and discussions from recent research, which reveal promising developments in the use of AI for BD management. Studies suggest that AI-powered tools can enable more proactive and personalized care, improving treatment outcomes and reducing the burden on healthcare professionals. AI’s ability to analyze data from wearable devices, smartphones, and even social media platforms provides valuable insights for early detection and more dynamic treatment adjustments. In Table 1, we provide a quick overview of the Introduction section, namely, the connection between BD and the emerging role of AI in its management, highlighting how AI can improve mood prediction, personalize treatment, and provide continuous support, while also addressing the challenges and future directions of AI-driven mental health care.

## 2. Discussion

In this chapter, we will also examine the practical challenges associated with integrating AI into BD management. One significant challenge is the need for large-scale, diverse data sets to train AI models that can accurately predict mood episodes across different populations. By discussing these challenges, we will explore how the integration of AI into clinical practice can be hindered by data limitations and biases, and why these concerns must be addressed to ensure that AI tools are effective for all patients [21].

Another critical area of focus will be on personalization in AI-driven treatment plans. One of the most promising aspects of AI is its ability to tailor interventions to the individual’s unique characteristics, including their genetic makeup, lifestyle factors, and personal history with BD. Personalized treatment plans can offer more effective care, and by discussing this, we will highlight why this approach is not only innovative, but essential for improving patient outcomes and minimizing adverse effects [23].

Furthermore, we will explore AI-powered mood tracking systems and how they can enhance the real-time monitoring of mood fluctuations. Traditional methods often rely on patient self-reports, which can be inconsistent or inaccurate, whereas AI can continuously track data from wearable devices, smartphones, and social media. We will discuss the significance of real-time monitoring in managing BD and why it offers a more proactive, responsive approach to care, preventing episodes before they escalate.

Finally, we will touch upon the future directions of research in AI for BD management, exploring the potential for further advancements and improvements. The field is still in its early stages, and future research could expand AI’s capabilities, making it an indispensable part of BD care. We will conclude by emphasizing the importance of ongoing research in refining AI algorithms, improving patient outcomes, and addressing ethical concerns to create an inclusive, accessible, and effective treatment landscape for BD [22].

By discussing these topics, we aim to provide a clear and thorough understanding of AI’s potential in BD management, while also critically evaluating its limitations and challenges.

### 2.1. Revealing the Complexities of Bipolar Disorder: Understanding the Challenges

BD is a chronic mental health condition that impacts millions globally, causing extreme mood fluctuations between manic and depressive episodes. The condition creates significant challenges not only for the individual experiencing the mood swings, but also for healthcare providers attempting to provide consistent care. Traditional treatments—such as medications and therapy—have been effective for many individuals but often fail to address the underlying causes of these mood changes. The unpredictable nature of the disorder complicates the treatment process, as the severity and duration of mood episodes can vary widely. In some cases, BD patients may experience rapid cycling, making it difficult for healthcare providers to maintain an effective long-term strategy [1,24,25].

Moreover, the societal stigma surrounding mental health disorders such as BD adds another layer of complexity. Many individuals with BD may delay seeking help, or they may not receive the necessary support from family, friends, or colleagues, worsening their overall health outcomes. It is crucial to identify how such complexities can be better managed and how advancements in healthcare can offer solutions to reduce the impact of these challenges [1,24,25].

The growing research into the role of AI in mental health management, particularly BD, brings the hope of a more precise and personalized treatment approach. By leveraging data from various sources, such as wearable devices, smartphones, and even social media, AI may provide better insights into mood fluctuations and help predict future episodes. The promise lies in offering proactive care that is tailored to an individual’s unique needs rather than simply reacting to symptoms as they occur.

### 2.2. Identifying the Rise of AI in Mental Health: A Game-Changer for BD Management

Artificial intelligence (AI) has emerged as a key tool for addressing the challenges associated with managing bipolar disorder (BD). In recent years, AI technology has demonstrated significant potential in transforming how mental health conditions are diagnosed, treated, and monitored. Traditionally, BD management relies on a combination of medications, therapy, and lifestyle adjustments, but these approaches are often reactive, intervening only after an episode has occurred. AI, in contrast, introduces a proactive element by leveraging real-time data from wearable devices, smartphones, and social media to detect patterns in behavior and mood. Machine learning algorithms analyzing these data can predict manic or depressive episodes, allowing for early clinical intervention and reducing the severity and duration of episodes.

AI integration into psychiatric care is still in its early stages, but several AI-driven systems have shown promising results in real-world clinical settings. For instance, AI-powered decision-support tools are being implemented in psychiatric hospitals to assist clinicians in diagnosing mood disorders, predicting relapse risks, and optimizing treatment plans. Machine learning algorithms analyzing electronic health records (EHRs) have been used to identify high-risk patients for early intervention, reducing hospitalization rates. Additionally, AI-driven mood monitoring apps and digital phenotyping tools are increasingly utilized in clinical environments to track patient symptoms and detect early warning signs of mood episodes. A notable example is the use of AI-enhanced fMRI analysis in schizophrenia research, which has improved the identification of biomarkers and enabled more personalized treatment strategies [6].

The ability of AI to analyze vast amounts of behavioral data represents a breakthrough in mental health management, helping move beyond the limitations of patient self-reports and providing more accurate, objective assessments of mood fluctuations. Moreover, AI-driven tools support clinicians in making informed decisions regarding medication adjustments and therapeutic interventions, offering a more refined and efficient approach to BD management. However, widespread adoption remains limited due to challenges such as regulatory approval, data privacy concerns, and the need for further clinical validation across diverse populations. As AI technology continues to evolve and demonstrate efficacy, its role in psychiatric hospitals and BD management is expected to expand, enhancing the precision and accessibility of mental health care [3,26].

### 2.3. Defining the Role of AI-Powered Mood Tracking: Enhancing Real-Time Monitoring

Traditional mood tracking methods often rely on patient self-reporting, which can be subjective and inconsistent. For individuals living with BD, recognizing early signs of mood shifts is crucial to preventing full-blown episodes. AI-powered mood tracking systems offer a more objective, continuous approach to monitoring mood fluctuations, using data from wearables, smartphones, and digital health apps to track a variety of behavioral metrics.

For example, sleep patterns, physical activity, speech speed, and social interactions are all indicators that can help detect subtle mood changes before they become significant. By applying machine learning algorithms to these data, AI can accurately identify early symptoms of both manic and depressive episodes. These predictive insights allow for timely interventions, enabling clinicians to adjust treatment plans or suggest behavioral changes before the episode fully develops.

This kind of real-time monitoring offers many advantages over traditional mood tracking, providing patients and clinicians with a more proactive and informed approach to managing BD. Additionally, the continuous data stream allows individuals to gain a deeper understanding of their mood patterns, giving them more agency in managing their own condition. Real-time feedback from AI-powered mood trackers can also support patients in identifying triggers, better managing stress, and engaging in self-care practices, which can ultimately lead to better long-term outcomes [27].

### 2.4. Exploring Personalized Treatment Plans: AI’s Role in Tailoring Therapy to Individual Needs

Personalized treatment is one of the most promising advancements in healthcare and it is especially beneficial for those with BD. Each individual’s experience with BD is unique, and a tailored treatment approach can lead to better outcomes than the standard, one-size-fits-all model. AI is well-positioned to play a significant role in creating personalized treatment plans for BD patients. By analyzing a combination of behavioral data, genetics, medical history, and even environmental factors, AI systems can generate treatment recommendations that are specifically suited to the individual.

For example, machine learning algorithms can assess which medications are most likely to work for a particular patient based on their genetic makeup and prior treatment responses. This reduces the trial-and-error approach often associated with psychiatric medication, ensuring more efficient and effective treatment. Furthermore, AI can continually monitor patient progress and make real-time adjustments to treatment plans, ensuring that care remains personalized and effective over time [28].

By taking a holistic approach—factoring in lifestyle choices, genetic predisposition, and behavioral data—AI can offer BD patients a comprehensive treatment plan that optimizes their chances for long-term stability. The ability to constantly refine treatment based on ongoing data allows for a dynamic, responsive approach, which contrasts sharply with the static, sometimes rigid treatment plans of traditional care models [29].

### 2.5. Resolving the Dilemma of Emotional Support Gaps: AI Chatbots Filling the Void

An ongoing challenge for individuals with BD is the lack of consistent emotional support between therapy sessions. Given the unpredictable nature of mood fluctuations in BD, there are often gaps when patients experience intense emotional distress but cannot access immediate help. AI-driven chatbots have the potential to fill this gap, providing real-time, personalized emotional support at any time of the day or night [30].

These AI chatbots are designed to simulate human conversation and offer a range of coping strategies based on the user’s current emotional state. For example, if a patient is exhibiting signs of mania, the chatbot might guide them through grounding exercises or mindfulness techniques to help manage symptoms. During depressive episodes, the chatbot can offer encouragement, suggest healthy behaviors, or help the patient connect with their treatment plan [11].

While these chatbots cannot replace human therapy, they offer valuable supplementary support, especially during moments of crisis. By continuously collecting data on patient interactions, AI chatbots can also provide clinicians with valuable insights into the patient’s emotional state, which can inform future treatment adjustments. Chatbots provide a non-judgmental space where patients can express their emotions freely, making it easier for them to reach out when they need help the most [31].

### 2.6. Discussing AI in Cognitive Behavioral Therapy (CBT): Enhancing Traditional Approaches

CBT has long been considered one of the most effective therapeutic approaches for individuals with BD. However, traditional CBT sessions are structured and occur at fixed intervals, leaving patients without immediate support during times of emotional distress. AI can significantly enhance the CBT process by offering real-time feedback between therapy sessions [13].

AI-driven platforms can allow patients to input their thoughts, behaviors, and emotional responses throughout the day. The system can analyze these data to identify cognitive distortions and offer suggestions for reframing negative thought patterns. By providing immediate, automated feedback, AI ensures that patients receive continuous support in their daily lives, helping them stay on track with their therapeutic goals. Furthermore, AI-powered CBT platforms can track a patient’s progress, offering personalized insights and suggestions to inform future therapy sessions.

By integrating AI into CBT, treatment becomes more dynamic and responsive, providing patients with the tools they need to manage their condition outside of scheduled sessions. This continuous learning process makes CBT more accessible and impactful, particularly for BD patients who may need frequent guidance in coping with the challenges of their condition [32].

### 2.7. Exploring the Ethical Implications of AI in BD Treatment: Striking the Balance

The integration of AI into the treatment of bipolar disorder (BD) introduces important ethical considerations that must be addressed to ensure the technology is used responsibly. One of the most significant ethical dilemmas is related to patient privacy and the handling of sensitive mental health data. AI systems rely heavily on personal data from wearables, apps, and social media platforms to track mood changes and predict episodes. Ensuring that these data are protected and used in a manner that respects patient confidentiality is crucial [18]. Moreover, there are concerns about the potential for AI systems to make decisions that may not always align with the nuances of individual patient needs. While AI can provide valuable insights and recommendations, it is important that human clinicians remain at the center of decision making. Over-reliance on AI could lead to a depersonalized approach to care, where patients may feel that their unique circumstances and experiences are not fully considered [33].

In addition to privacy and accuracy concerns, patient trust in AI-driven interventions remains a key challenge. For many BD patients, using AI-driven tools in treatment raises questions about the technology’s reliability and its ability to accurately understand and respond to their emotions and experiences. Studies show that while some patients view AI as a useful tool for managing their condition, others remain cautious or resistant, expressing concerns about the lack of human interaction and emotional support that they value in traditional care [34,35]. Patients’ engagement with AI technologies is significantly influenced by the perceived authenticity of these tools and their trust in the technology. Therefore, it is crucial for clinicians to foster transparency and clear communication about how AI works, what data it collects, and how it can support the patient’s treatment plan, ensuring that AI serves as a complement to, rather than a replacement for, human care.

Another ethical challenge is the risk of bias in AI algorithms. If the data used to train AI systems are not representative of diverse populations, there is a risk that certain groups may be underserved or misrepresented by the technology. Ensuring that AI models are trained on a broad, inclusive dataset is vital to preventing these biases from influencing treatment decisions [36].

Ultimately, striking a balance between technological advancement and ethical responsibility is key to the successful integration of AI in BD treatment. While AI has the potential to revolutionize mental health care, its ethical implications must be carefully considered to protect patient autonomy, privacy, and well-being.

### 2.8. Identifying the Limitations of AI in Mental Health: Acknowledging the Gaps

Despite the promising applications of AI in managing bipolar disorder (BD), it is crucial to recognize its limitations. AI models have significantly enhanced psychiatric assessments by improving diagnostic precision, early detection, and personalized treatment strategies. Unlike traditional evaluations that rely on clinician interpretation and patient self-reports, AI leverages real-time data from speech patterns, wearable devices, and neuroimaging to identify subtle mental health markers. However, the effectiveness of AI depends heavily on data quality, which can be inconsistent or incomplete due to reliance on patient self-reports, smartphones, and wearable devices, potentially reducing the accuracy of predictions and treatment recommendations [37]. Furthermore, while AI excels at detecting patterns and trends, it struggles to capture the complexity of human emotions, personal relationships, and lived experiences, limiting its ability to provide a truly comprehensive understanding of a patient’s condition.

Another challenge lies in determining which AI approaches—such as deep learning, rule-based systems, or hybrid models—yield the best results in BD management. Deep learning models, particularly neural networks, can process vast datasets and uncover intricate patterns that might be overlooked by human clinicians, offering high accuracy in mood prediction. However, these models often lack interpretability, making it difficult to understand their decision-making processes. In contrast, rule-based AI systems offer greater transparency, but may not adapt well to the complexities of individual patient cases. Hybrid approaches that integrate machine learning with clinician input may offer a balanced solution, but further research is needed to validate their long-term effectiveness.

Additionally, AI’s increasing role in psychiatry raises concerns about diminishing the human aspect of mental health care. While AI-powered tools can assist in detecting mood episodes and relapse risks, they lack the empathy, nuanced clinical judgment, and therapeutic alliance essential for effective psychiatric treatment [38]. Ethical considerations, including data privacy, algorithmic biases, and accessibility, must also be addressed to ensure AI-driven tools enhance rather than undermine psychiatric care. As AI continues to evolve, it should be viewed as a complementary tool rather than a replacement for human expertise, ensuring that BD treatment remains both technologically advanced and patient-centered [39,40,41].

### 2.9. Defining the Role of Clinicians in an AI-Driven Mental Health Landscape

As AI becomes an increasingly important part of managing BD, the role of clinicians in the mental health landscape is evolving. AI has the potential to enhance clinical decision making by providing more accurate, data-driven insights into patient behavior and treatment responses. However, clinicians remain at the heart of the therapeutic process, and their expertise is essential in interpreting and applying AI-generated recommendations.

AI can assist clinicians by offering real-time data on a patient’s mood, behaviors, and treatment progress. This allows for more personalized and dynamic treatment plans, as AI can predict potential mood episodes and recommend interventions. Clinicians can then assess these recommendations, consider the patient’s unique circumstances, and make the final decisions regarding treatment [18].

Additionally, clinicians play a vital role in ensuring that AI is used ethically and responsibly. They must be vigilant about patient privacy, consent, and the potential risks of relying too heavily on AI in mental health care. By maintaining a strong therapeutic relationship and offering human insight, clinicians can help bridge the gap between technological innovation and compassionate care [42].

Ultimately, the future of BD treatment lies in the collaboration between AI technology and human expertise. AI can provide valuable insights, but clinicians remain indispensable in providing the empathy, understanding, and personalized care that patients need [18,37].

### 2.10. Discussing the Future of AI in BD Treatment: Opportunities and Challenges Ahead

The future of AI in BD treatment holds immense potential, but it also presents several challenges. As AI technology continues to evolve, it is likely that it will become more integrated into the management of BD, offering new tools for diagnosis, treatment, and ongoing monitoring [37,43].

One exciting opportunity is the potential for AI to improve the early detection of BD. By analyzing patterns in behavior, speech, and even facial expressions, AI systems may be able to identify mood shifts before they become significant, allowing for earlier interventions. This could lead to a reduction in the frequency and severity of mood episodes, improving the overall quality of life for individuals with BD [43].

Additionally, AI could enhance the personalization of treatment. By analyzing vast amounts of data, AI could identify the most effective treatments for each individual based on their specific symptoms, genetic factors, and treatment history. This tailored approach would reduce the reliance on trial-and-error strategies and offer more precise, effective care.

However, the widespread adoption of AI in BD treatment also presents challenges. One key concern is the accessibility of AI-powered tools. While AI has the potential to revolutionize mental health care, ensuring that these tools are accessible to all individuals, regardless of socioeconomic status or geographical location, is essential. Additionally, addressing concerns related to data privacy and bias in AI algorithms will be crucial in ensuring that the technology is used ethically and responsibly [44].

As AI continues to advance, its role in BD treatment will likely grow, offering new opportunities for more effective, personalized care. However, its integration into the mental health landscape must be approached thoughtfully, with careful attention to both the opportunities and challenges it presents.

### 2.11. Resolving the Dilemma of Accessibility: Making AI Tools Available for All

While the potential of artificial intelligence (AI) in managing bipolar disorder (BD) is undeniable, one of the key dilemmas in implementing AI-driven solutions is ensuring accessibility for all individuals who could benefit. Accessibility is a multifaceted issue, encompassing factors such as cost, availability, and technological literacy [45].

The cost of AI-powered mental health tools remains a significant challenge, particularly for low-income individuals. While AI technologies have shown great promise in enhancing mental health care, including mood tracking and therapeutic support, these tools often come with subscription fees or other costs that may be prohibitively expensive for many patients. Moreover, the affordability of AI systems for widespread clinical use is another concern. While some AI-driven platforms demonstrate clinical effectiveness, their high costs limit their availability and accessibility. To ensure equitable access to these technologies, it is crucial to explore ways to subsidize or provide free access to AI tools, particularly for vulnerable populations who may lack the financial means to benefit from them. Furthermore, the cost-effectiveness of these systems in comparison to traditional methods needs to be considered, as ensuring affordability is vital to their integration into routine clinical practice. Addressing these barriers is essential for the successful and widespread adoption of AI-powered mental health care [34,35,46].

In addition to cost, technological literacy is another critical factor. Not all individuals, particularly older adults or those in rural areas, have the digital skills or access to the technology needed to take advantage of AI tools. To bridge this gap, educational programs and community outreach efforts are needed to help individuals understand how to use AI-driven platforms effectively. Additionally, ensuring that AI tools are designed to be user-friendly and accessible to a wide range of users, regardless of their technical expertise, is essential for increasing adoption [47].

Finally, geographical accessibility is another consideration. Many mental health services, including those that use AI, are concentrated in urban areas, leaving rural and remote communities with limited access to these innovations. To address this issue, telemedicine platforms and mobile health apps that incorporate AI could play a key role in making mental health care more accessible to individuals in underserved areas [47,48].

Ensuring the widespread accessibility of AI tools in BD treatment requires a concerted effort from both technology developers and policymakers to reduce barriers related to cost, literacy, and geography. Only through these efforts can AI-driven solutions reach the individuals who need them most, helping to create a more equitable mental health care system for all.

At the very end of the discussion, we present Table 2, summarizing the key discussion points in the provided chapter. This overview highlights the major topics, their key aspects, and their significance in the context of AI integration in BD management. This table provides a concise overview of the major points discussed in the chapter, helping to summarize the key aspects of AI’s role in managing BD and the associated challenges (Table 2).

## 3. Conclusions

In conclusion, we have found that AI holds significant potential in transforming the management of BD. Regarding our primary objective, we conclude that AI tools, including mood tracking devices and machine learning algorithms, show promise in monitoring mood fluctuations, predicting manic or depressive episodes, and creating personalized treatment regimens. These technologies could revolutionize how BD is managed by providing real-time insights and more tailored interventions.

In line with our secondary objective, we reviewed recent research that supports these findings, revealing that AI-powered tools offer the possibility of more proactive and personalized care. By analyzing data from wearable devices, smartphones, and even social media platforms, AI enables the early detection of mood shifts and dynamic treatment adjustments. This can enhance treatment outcomes and reduce the burden on healthcare professionals.

Ultimately, while AI’s application in BD management is still in its early stages, we conclude that it carries transformative potential for improving both the quality of care and the efficiency of treatment delivery. However, further research and development are essential to fully realize AI’s capabilities in supporting BD patients and optimizing therapeutic strategies. 

## Figures and Tables

**Table 1:** Overview of the Introduction section.

Section	Summary
Introduction to Bipolar Disorder	BD is a chronic mental health condition characterized by extreme mood swings, including manic and depressive episodes. These mood fluctuations severely affect daily life and present significant challenges for both individuals and healthcare providers. Traditional treatments, such as medication and therapy, often fail to predict or prevent mood episodes, leading to the search for more innovative solutions.
AI in BD Management	AI offers a promising advancement in BD management. AI has the potential to predict mood episodes, track behaviors in real-time, and tailor treatment plans based on individual needs. Through machine learning algorithms and real-time data from wearable devices and social media, AI can provide insights that help identify early signs of manic or depressive episodes, enabling proactive and personalized care.
AI-Powered Mood Prediction	AI can predict mood fluctuations by analyzing patterns in data such as sleep, activity levels, and social media usage. By detecting early symptoms of manic or depressive episodes, AI allows healthcare providers to intervene before episodes escalate, offering a more proactive, data-driven approach to care.
Personalized Treatment Plans	AI enhances the personalization of BD treatment by analyzing genetic, behavioral, and environmental data. This helps create tailored treatment regimens, adjusting medications and therapies based on real-time responses to improve outcomes and reduce the trial-and-error approach commonly used in traditional care.
Real-Time Support and Monitoring	AI provides continuous support through real-time mood tracking via wearable devices and mobile apps. By monitoring patients’ behaviors, AI can offer immediate feedback and suggest coping strategies during critical moments, bridging gaps between therapy sessions and ensuring ongoing care.
Predictive Models for Relapse Prevention	AI-driven predictive models analyze patterns in behavioral data to forecast mood episodes before they occur, enabling early interventions. These predictive models help reduce the severity of episodes, prevent hospitalization, and improve long-term treatment outcomes by proactively adjusting medications or suggesting lifestyle changes.
Challenges and Ethical Considerations	The integration of AI into BD management raises ethical concerns, including patient privacy, consent, and the accessibility of AI-driven tools. Ensuring data security, transparency, and the complementary role of AI in human care is crucial for successful adoption. Additionally, AI should be accessible to diverse populations to maximize its potential benefits.
Future Directions and Research Gaps	Research on AI in BD management is still evolving. Large-scale studies and continuous refinement of AI algorithms are needed to validate their effectiveness and explore how AI can best complement traditional therapies. Ethical implications and patient outcomes need to be evaluated as AI tools become more prevalent in mental health care.
AI’s Transformative Potential in BD Care	AI offers transformative potential in BD care, enabling continuous mood tracking, personalized treatment, and proactive management. Despite challenges related to ethics and accessibility, AI’s integration into BD treatment can improve patient outcomes, enhance treatment efficacy, and revolutionize the landscape of mental health care.

**Table 2:** Overview of key discussion points on AI integration in BD management.

Topic	Key Discussion Points	Significance
2.1. Revealing the Complexities of Bipolar Disorder	Chronic mental health condition with mood fluctuations between manic and depressive episodes. Challenges include unpredictable episodes, rapid cycling, and societal stigma.	Highlights the complexity of BD and the need for innovative solutions in treatment.
2.2. Identifying the Rise of AI in Mental Health	AI’s potential to proactively manage BD by analyzing data from wearables, smartphones, and social media to predict mood episodes.	AI’s predictive capability transforms BD treatment, offering proactive care rather than reactive intervention.
2.3. Defining the Role of AI-Powered Mood Tracking	AI systems track mood fluctuations via wearables and apps, offering continuous, objective monitoring. Machine learning algorithms can predict early symptoms of mood shifts.	Provides a more accurate and consistent method of tracking mood fluctuations, enabling early intervention and more personalized care.
2.4. Exploring Personalized Treatment Plans	AI uses behavioral data, genetics, and medical history to tailor treatment plans for BD patients. Real-time adjustments are possible based on continuous monitoring.	Personalization leads to more effective treatments and reduces trial-and-error methods in psychiatric care.
2.5. Resolving the Dilemma of Emotional Support Gaps	AI chatbots offer real-time emotional support for BD patients between therapy sessions, providing coping strategies and insights into emotional states.	Helps bridge emotional support gaps and allows patients to receive immediate assistance, reducing distress during crises.
2.6. Discussing AI in Cognitive Behavioral Therapy (CBT)	AI enhances CBT by providing real-time feedback and analyzing thoughts and behaviors, which it offers continuous support outside of scheduled sessions.	Enhances the effectiveness of CBT by offering ongoing support and personalized feedback, improving patient engagement and outcomes.
2.7. Exploring the Ethical Implications of AI in BD Treatment	Key ethical concerns include patient privacy, AI’s potential biases, and over-reliance on technology. Human clinicians must remain at the center of decision making.	Ensures AI use respects patient autonomy, privacy, and diversity, balancing innovation with ethical responsibility.
2.8. Identifying the Limitations of AI in Mental Health	AI relies on high-quality, consistent data and may not fully capture the complexities of human behavior. It cannot replace the human empathy and understanding provided by clinicians.	Acknowledges AI’s limitations and the importance of combining it with human expertise in BD care.
2.9. Defining the Role of Clinicians in an AI-Driven Mental Health Landscape	Clinicians remain essential in interpreting AI insights, maintaining patient relationships, and making final treatment decisions. AI assists, but does not replace clinicians.	Clinicians’ expertise ensures that AI-generated recommendations are applied effectively, preserving the human element in treatment.
2.10. Discussing the Future of AI in BD Treatment	AI’s potential for early detection of BD, personalized treatment strategies, and the challenges of accessibility, data privacy, and bias in algorithms.	The future of BD treatment lies in advancing AI capabilities, but challenges such as equity, accessibility, and ethics need to be addressed.
2.11. Resolving the Dilemma of Accessibility	Barriers to AI adoption include cost, technological literacy, and geographic location. Solutions include subsidized access and educational programs to improve digital literacy.	Ensures that AI-powered tools are accessible to all individuals, particularly underserved populations, promoting a more equitable healthcare system.

## Data Availability

Data are contained within the article.

## Abbreviations

The following abbreviations are used in this manuscript:

BD	Bipolar disorder
AI	Artificial Intelligence
CBT	Cognitive behavioral therapy
